# Applying Heart Rate Variability to Monitor Health and Performance in Tactical Personnel: A Narrative Review

**DOI:** 10.3390/ijerph18158143

**Published:** 2021-07-31

**Authors:** Mark D. Stephenson, Andrew G. Thompson, Justin J. Merrigan, Jason D. Stone, Joshua A. Hagen

**Affiliations:** Rockefeller Neuroscience Institute, West Virginia University, Morgantown, WV 26505, USA; andrewg.thompson@hsc.wvu.edu (A.G.T.); justin.merrigan@hsc.wvu.edu (J.J.M.); jason.stone1@hsc.wvu.edu (J.D.S.); joshua.hagen@hsc.wvu.edu (J.A.H.)

**Keywords:** heart rate variability, HRV, autonomic nervous system, military, human performance, physiological monitoring

## Abstract

Human performance optimization of tactical personnel requires accurate, meticulous, and effective monitoring of biological adaptations and systemic recovery. Due to an increased understanding of its importance and the commercial availability of assessment tools, the use of heart rate variability (HRV) to address this need is becoming more common in the tactical community. Measuring HRV is a non-invasive, practical method for objectively assessing a performer’s readiness, workload, and recovery status; when combined with additional data sources and practitioner input, it provides an affordable and scalable solution for gaining actionable information to support the facilitation and maintenance of operational performance. This narrative review discusses the non-clinical use of HRV for assessing, monitoring, and interpreting autonomic nervous system resource availability, modulation, effectiveness, and efficiency in tactical populations. Broadly, HRV metrics represent a complex series of interactions resulting from internal and external stimuli; therefore, a general overview of HRV applications in tactical personnel is discussed, including the influence of occupational specific demands, interactions between cognitive and physical domains, and recommendations on implementing HRV for training and recovery insights into critical health and performance outcomes.

## 1. Introduction

The health, well-being, and preparedness of tactical personnel are crucial to mission success [1]. In tactical settings, Human Performance Optimization (HPO) programs focus on enhancing and sustaining the individual’s ability to perform occupational tasks over time. There are several strategic focus areas that contribute to HPO of tactical personnel, to include physical fitness, health, nutrition, cognitive, and psychosocial. Inherently, military training and operations have remarkably high physical and mental demands that necessitate exceptional levels of fitness [2,3]. To achieve such extreme levels of prowess, bouts of higher training intensities and volume overload are required [4], subsequently allowing the operator to develop hypersensitivity to chronic fatigue and overtraining [5]. However, incomplete recovery from training may also lead to chronic fatigue, which inevitably hinders operator preparedness [6]. Fortunately, scientifically validated approaches in sport and exercise sciences provide the basic concepts and principles for applications of monitoring training loads in tactical settings [7].

The need for accurate, meticulous, and effective monitoring of biological adaptations and systemic recovery is certainly critical for all domains under HPO [8]. In addition, the practice of self-monitoring affords the individual operator a platform for personal insights and real-time biofeedback [9,10], enhancing the opportunity to facilitate in/exteroception capacity and regulation capability [11], implement necessary training modifications or lifestyle adjustments, and mitigate unwanted adaptations (e.g., diminished power output, negative mood states, increased sympathetic tone, skill/knowledge decay) [12]. Monitoring training loads (TL), which can be segmented into external and internal training loads, can be used to manage cumulative TL on an individual and group level [13]. External loads, typically measured by global positioning systems, accelerometers, transducers, or gyroscopes, represent physical work being incurred by the individual, such as, total distance covered, high impacts, bouts of accelerations and decelerations, repetitions, sets, and intensities of resistance training [13,14]. Meanwhile, the internal loads represent the physiological costs incurred by exposure and production of forces during external loads. Internal loads may be monitored objectively through laboratory testing (e.g., blood, blood lactate, saliva, hormone, and metabolic measure) and wearable portable physiological measuring devices (e.g., heart rate monitor), while subjective measures can be monitored by use of rating scales (e.g., rate of perceived exertion (RPE), fatigue scale, sleep quality, etc.) and subjective questionnaires (e.g., Pittsburgh Sleep Quality Index) [7,13,14,15,16,17]. Individuals respond differently to given external loading for numerous reasons (e.g., current fitness levels, genetics, sleep hygiene), and thus it is particularly important to monitor internal loads. In tactical populations, global positioning systems and accelerometers have been used to quantify external loads while laboratory testing, wearable physiological sensors, and subjective measures have been commonly used to assess internal loads [14].

In HPO programs, the utilization of heart rate variability (HRV) provides a non-invasive, practical method for assessing an individual’s response to internal load [5,18]. The ability of HRV to afford immediate insight on individual adaptability to environmental demands (i.e., training load) [5,19,20,21,22] is particularly important for the strenuous nature of military training [14]. Without proper monitoring and reactive/proactive programming, intense training cycles can lead to maladaptive outcomes (e.g., overtraining or injury) thereby compromising preparedness for occupational tasks [2,23,24,25]. Ultimately, the use of HRV provides insights on the neurocardiac function of the autonomic nervous system (ANS) [22,26], permitting inferences into overall performer preparedness/workload status and resource availability/efficiency [15,22,27,28]. To ascertain the value of employing HRV in HPO programs, the framework and context pertaining to ANS architecture must be understood. Although literature exists regarding the ANS and HRV methods, limited research has employed HRV in tactical populations. A meta-analysis performed by Tomes and colleagues [29] concluded that HRV was an effective tool for measuring health and performance in tactical personnel. However, despite these findings, literature is lacking that describes various field applications and actionability of HRV data on performance recovery and sustainment. Further, due to the innate uniqueness, there may be special considerations for employing HRV monitoring in tactical settings, which require further investigation. Thus, the purpose of this narrative review is to (1) review the underlying foundations behind the use and limitations of HRV, (2) identify some of the appropriate applications of HRV metrics in tactical settings, and (3) explore the relationship between HRV and stress, occupational performance, cognitive performance, and recovery in tactical environments.

## 2. Autonomic Nervous System (ANS)

The ANS influences body functions by regulating systemic resource allocation to maintain homeostasis and accomplish goal specific actions in the face of fluctuating internal and external environmental stimuli [30,31]. Practically, it moderates physiological and psychological preparations for and recovery from acute and chronic workloads. This regulatory system’s stability and responsiveness is critical for meeting the unpredictable and strenuous occupational demands imposed on military personnel [32]. Moreover, many tactical performance constructs can be directly linked to the efficiency and effectiveness of ANS-modulated systemic responses, such as: endocrine profiles supportive of passing intense selection events [6,24,25]; plasticity to adapt with progressive training overload across critical functional areas (e.g., cardiovascular, musculoskeletal, cognitive) in the development and maintenance of peak operational capabilities [2,3,23,33]; emotional awareness and regulation to sustain before, during, and after a firefight [11,34]; motor control resiliency required to execute mission essential tasks [35]; and the overall system’s ability to recover, repair, and improve from various stimuli [32]. Collectively, the ANS is implicated in nearly every aspect of human behavior, with innate links to health and performance [36]. The direct application to high stress, high-stakes vocations only strengthens this connection and elevates the importance of understanding the functional physiology involved.

**Figure 1:**
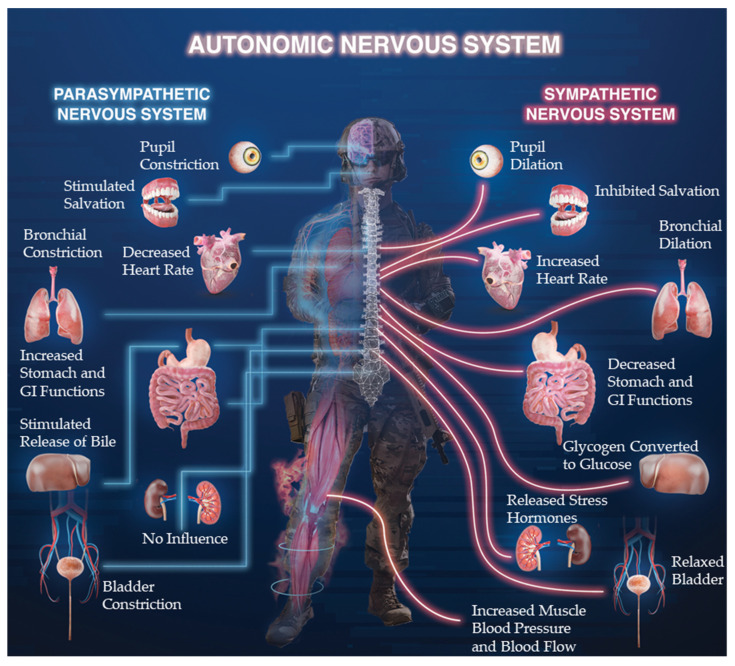
Example layout of the relations between various organs throughout the body and the Autonomic Nervous System.

The primary ANS (excluding enteric) is comprised of the sympathetic nervous system (SNS) and parasympathetic nervous system (PNS), which are associated with arousal (often perceived as stress) or relaxation, respectively [5,37,38]. Variations in cardiac dynamics (e.g., HRV) are the result of intricate interactions between the SNS and PNS [5,38]. The ANS’s influence on the cardiovascular system is regulated by a complex network of brain centers (e.g., limbic system, hypothalamus, and medulla) and neural communication pathways (e.g., brainstem and spinal cord) [11]. The medulla integrates sensory input and regulates parasympathetic outflow to help control cardiac modulation in response to arousal, stress, and task demands [5,11,31,38,39]. Primarily, the ANS controls cardiac function through its innervation of the sino-atrial (SA) node (i.e., heart’s pacemaker) and atrioventricular (AV) node via the right and left vagus nerves, respectively [5,39,40,41]. Resultantly, the ANS regulates heart rate, cardiac timing, contractility, and conduction velocity (i.e., mechanisms that facilitate resource delivery). The sympathetic and parasympathetic divisions are often complementary, whereas the SNS generally increases heart rate and vasoconstriction, the PNS generally decreases heart rate and causes vasodilation (Figure 1) [5,38,39]. During fight or flight environmental demands, the need to deliver metabolic substrate for movement overrides the “resting state” variability induced by SA-node conduction delay [42]. However, the SNS and PNS do not always increase and decrease activation in a reciprocal manner, especially during high-stress skill execution [5,35].

Since the ANS is intimately connected with other psychological and physiological systems throughout the body, quantifying responsiveness can provide vital information regarding functional occupational performance capacity or adaptions during/after exercise and training iterations [38]. This is particularly important to understand in tactical personnel, who are frequently or unpredictably exposed to high acute stressors, bouts of intensive physical activity, extreme environments, and varying occupational schedules (i.e., rotating night shifts, multi-day combative engagements), which may directly influence the cardiovascular system [43]. A schematic diagram of SNS and PNS components for various physiological systems is displayed in Figure 1. The acceleratory responses from the SNS, including elevated heart rates, muscular perfusion, bronchial dilation, and pupil dilation are useful for preparing the body’s response to situations requiring physical prowess and precise perceptual motor coupling, which are often noted in tactical settings. Chronically elevated SNS responses can have negative consequences, requiring PNS activation or SNS deactivation to ensure adequate recovery from high intensity events and promote overall health and well-being (i.e., reducing symptoms of burnout, post-traumatic stress disorder, or depression) [2,6,32]. Further, sympathetic responses can impede activity and efficiency in specific brain regions, such as the pre-frontal cortex, which is responsible for higher level executive functions, and the motor cortices [34,35,44]. Inhibition of the pre-frontal cortex can negatively affect attention, emotion, and decision-making, while further impeding the fine motor skills needed for occupational tasks of tactical personnel [11,12,34,44,45]. In tactical populations, measuring and monitoring ANS responses, such as HRV, will likely be useful for informing skill efficiency and expertise, individualized training programs, occurrence or likelihood of injury or illness, risks of overtraining, training and recovery statuses, and operational preparedness. An individual’s HRV may be described through numerous calculations of raw heart rate data signals, which are deconstructed to quantify the underlying signal components, resonant frequency bands, and glean more value, greater inference, and actionability in the underlying data.

## 3. Defining Heart Rate Variability (HRV) and Respective Metrics

The term HRV describes a series of mathematical calculations for modeling psychophysiological responses based on the time between consecutive heart beats, also known as beat-to-beat or inter-beat-intervals (IBI) [5,20,26,46,47,48,49]. IBI is the duration between R peaks from consecutive QRS complexes (i.e., ventricular depolarization) across the duration of a contextually relevant time series (i.e., NN or R-R) [5,50,51,52]. The IBI is typically measured with an electrocardiography (ECG) signal with data sampling frequencies from 250–1000 Hz. Higher sampling frequencies offer more robust data, which is especially important for capturing HRV metrics during dynamic movements (e.g., exercise, room-clearing) and complex cognitive processes (e.g., event based decision making) [5,30,38,50]. The stress (e.g., physical activity, psychological arousal) induced sympathetic activation and parasympathetic (vagal) withdrawal causes the R-R intervals to become shorter, more rapid, and less varied, resulting in a global decrease across most HRV metrics [5,20,22,53]. Generally, sympathetic activation increases HR and decreases IBI variability, while parasympathetic withdrawal decreases HR and increases IBI variability. Typically, PNS modulation occurs rapidly (e.g., <1 s) and possesses a short-lived response (<5–10 s), while the SNS’s response is slower (~5 s from stimulation onset to IBI modulation) and persists for longer durations (~10–30 s) [41]. Measuring and analyzing IBI variations across time via HRV metrics can provide quantitative insights on the culmination of psychophysiological responses and further detail a performer’s ability to handle internal and external environmental demands (i.e., recovery) [22,53].

**Figure 2:**
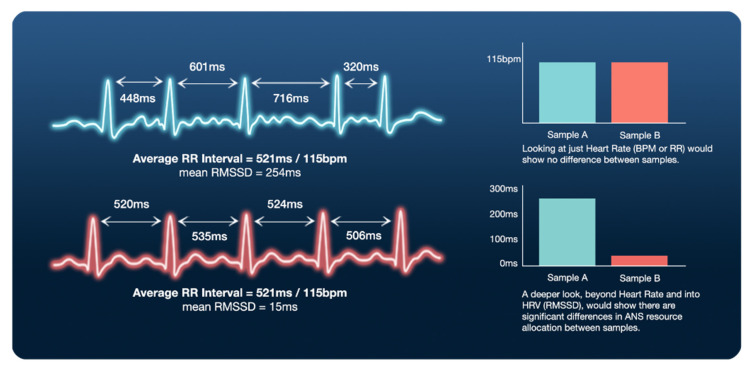
Example of R-R intervals in milliseconds (duration between R peaks from consecutive QRS complexes). Trace 2 (lower, in red) shows little/negligible heart rate variability (HRV). Trace 1 (upper, in blue) shows significant HRV. Data table on right exemplifies importance: average heart rate (HR as bpm) is identical, but when the underlying signal is teased apart, massive differences in root mean squared of successive differences (RMSSD ~ 17 fold) and can be used to infer significant differences in autonomic nervous system (ANS) resource allocation between the same samples.

With respect to training loads and recovery, the most frequently implemented and practically useful HRV parameters are analyzed using time- and frequency-domain methods [5,20,26]. These commonly used methods represent various ways to view the central tendency (i.e., mean, median, mode), variability, and distribution (i.e., standard deviation) of heart rate over time [5]. HRV parameters consider the average values and overall magnitudes of fluctuations to quantify control of heart rate over time. However, two individuals may have the same average R-R interval and heart rate (HR) responses to an event, but vastly different variability of R-R intervals (see Figure 2 for graphic example). The two most commonly accessible/used time-domain parameters are the standard deviation of the N-N intervals (SDNN) and the root mean square of the differences in adjacent N-N intervals (rMSSD). The SDNN represents a coarse quantification of HRV via autonomic regulation from sympathetic and parasympathetic inputs [21,54], while rMSSD represents parasympathetic activity [20,21,27,54,55]. Unlike SDNN, rMSSD is void of HR slow-wave components, resulting in minimal respiratory influence and a more accurate representation of parasympathetic activity [46,54].

Frequency domain metrics use mathematical transformations of R-R data to quantify the modulation of a given signal at specific frequency ranges (e.g., power spectral density bands): ultra-low frequency (ULF), very low frequency (VLF), low frequency (LF), and high frequency (HF) [26]. Compared to the time domain, frequency domain HRV metrics tend to offer more detail regarding the underlying signal, its component-based contributions, and their relationship to behavioral outcomes. In clinical settings, ULF (≤0.003 Hz) and VLF (0.0033–0.04 Hz) are often used during long-term recordings (≥24-h) [5,26]. However, athletic and tactical settings may not permit long recordings, thus, typically rely on more biologically relevant and accurate metrics during short-term recordings (≤10-min), such as LF (0.04–0.15 Hz) and HF (0.15–0.4 Hz) [5,56]. The HRV frequency domain metrics represent specific ANS branches [5,26,38]. The LF power is influenced by both SNS and PNS as well as baroreceptors (i.e., blood pressure control) [5,26]. In resting conditions (e.g., sleep), LF reflects the baroreflex, not cardiac sympathetic innervation [26,57], and may not accurately reflect HRV responses to training load and recovery. The HF power band directly reflects parasympathetic activity and can be influenced by the respiratory cycle [26,38] and cognitive workload demands (e.g., emotion, executive functioning, motor control) [11,58,59]. Low (and decreasing) HF is often associated with parasympathetic resource consumption (e.g., mental/physical task), distress (e.g., anxiety, worry), or poor resource efficiency (e.g., non-expertise, lack of recovery), and commonly leads to impaired physical or cognitive performance [23,26,60]. Insights on sleep quality, regeneration, and fatigue can also be obtained through analyzing frequency domain metrics during nocturnal HRV assessments [61,62].

The ratio of LF to HF is often used to represent sympathetic-parasympathetic balance [26,63,64]. This incorrectly assumes that LF solely reflects sympathetic activity, despite being produced by both the SNS and PNS [26,64]. Given those assumptions, a low LF/HF ratio would indicate parasympathetic dominance and a high LF/HF would indicate sympathetic dominance [26]. However, these relations may be influenced by body position, as LF is primarily produced by SNS in a supine position and PNS in a seated position [26,64]. These intricacies make it more difficult to use the LF/HF ratios in assessing training load and recovery [26]. Total power (TP) refers to the sum of all power spectral density components (ULF, VLF, LF, and HF), which reflects the total variance in heart rate pattern over recording durations [21,26,57,61,65,66,67,68]. Due to their reflection of general autonomic and parasympathetic activity, TP and HF are effective frequency-domain metrics for interpreting performance efficiency, training adaptation, and recovery/resiliency in the presence of physiological and psychological loads [5,26].

Consequently, in a resting state, increases in HF and TP can indicate desired training adaptations and complete systemic recovery, whereas a decrease in HF and TP typically indicates unwanted training effects, maladaptation, and incomplete recovery [26,34,35]. Mathematically, the modulation of HF to TP (HP/TP) may index the proportion of parasympathetic activity within the entire ANS frequency-domain spectrum. Overall, many HRV metrics exist (Table 1) and represent specific ANS domains, but their applicability and accuracy in specific settings need to be considered prior to their use. Typically, the three variables often considered the most meaningful and actionable are rMSSD, HF, and TP. The normative values for each of these components are dependent on many factors, such as age, gender, fitness, and various demographics. Therefore, context is required when drawing conclusions based on these HRV metrics. For all of these reasons, HRV metrics should be utilized as within-individual changes. Comparing one individual’s HF score to another’s would not provide any personalized insights. Overall, these HRV metrics will initially decrease under persistent conditions of high stress environment, poor sleep hygiene, poor nutrition, inadequate exercise, durations of isolation or harmful relationships. On the contrary, higher HRV values are often associated with positive health states and functionally beneficial adaptations, including cardiovascular fitness and resiliency to training loads or other various stressors.

## 4. Implementation and Analysis of Heart Rate Variability (HRV) Measures

### 4.1. How to Measure HRV

Field based HRV monitoring is considered user friendly, objective, and a particularly reliable tool, which can be easily used to manage training loads and facilitate proper recovery [5,53]. Additionally, HRV assessment and monitoring tools have become more affordable and practical to use [26,27]. With a continuously growing number of commercial devices capable of measuring and reporting HRV, it is first important to understand device accuracy [70], then equally necessary to understand their compatibility for use in the field with tactical populations. For example, the placement of the sensors, wiring requirements, duration of battery life, internal memory capacities, device durability (i.e., handling weather conditions and impacts), and screen displays (i.e., ability to be shut off on night missions) should all be considered when selecting devices. Detailed information on wearable sensors capable of measuring HRV in military settings can be found in a recent review article [71], which highlights the pros and cons of several devices. Overall, practical, commercially available wearable sensors tend to lack adequate battery life and internal memory for long duration recordings (24 h cycles), while clinical devices (3-12 lead electrocardiography) provide the most accurate results for long and short durations, particularly at rest [72]. Despite better signal to noise ratios, clinical electrocardiography (ECG) devices may still be susceptible to motion artifact or electrode impedance and lack practicality due to their bulkiness and wiring requirements, making them less suitable for tactical environments.

Practical devices, such as chest straps, clothing garments, and wrist or finger worn photoplethysmography (PPG), are unobtrusive, but their validity and reliability can be significantly impacted by the fit of the device, which should be “snug” to avoid missing data from disconnections [73]. The accuracy of PPG devices is also subject to motion artifact, skin pigment, tattoos, temperature, and pressure placed on the sensor [73]. At rest, certain PPG devices may be accurate [70] but reductions in accuracy are noted as intensity of exercise or movement variability increases [74]. Thus, PPG devices may be useful during sleep/resting stages or mild, continuous exercise, but may not accurately capture HRV during the intense events common to tactical populations. Yet, these devices prove useful for their ability to be conveniently worn on the wrist or finger, with little interruption to daily living, for overnight, upon awakening, or 24 h measurements. However, the utility of these devices in tactical settings requires further exploration. Chest strap devices (e.g., Polar H10) are typically utilized in a physical training application but can also accurately and reliably be used for HRV assessments [75]. We suggest that chest strap devices are most appropriate during tactically relevant physical maneuvers, while the specific manufacturer to use is subject to their level of accuracy during the intended testing events.

### 4.2. When to Measure HRV

**Figure 3:**
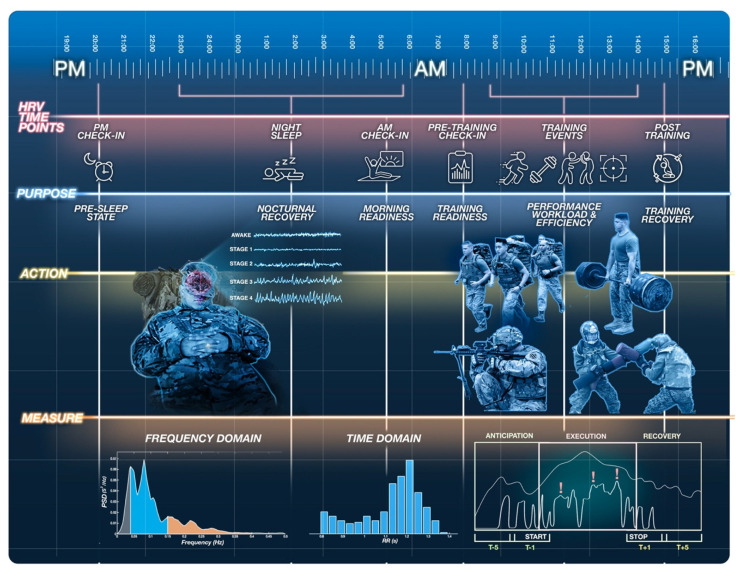
Opportunities within tactical populations for heart rate variability (HRV) collection throughout the day. Pre-sleep HRV assessments inform end of day status, daily resource consumption, and can quantify daily workload. During sleep, HRV provides insight on nocturnal recovery, homeostatic restoration, and daily readiness. Pre- and post-training HRV spot checks may inform acute preparedness for and recovery from training. During events, HRV can provide insights on performance workload and efficiency.

A measurement’s purpose dictates the appropriate schedule of data collection timepoints (e.g., time of day, duration, and frequency) [5]. Monitoring HRV may involve periodic resting measures and pre- and post-training assessments across key timepoints to capture autonomic resource distribution, consumption, and restoration before, during, and following specific task demands. Following training, the time required for HRV to return near baseline values may demonstrate the relative ability to recover from training sessions of various intensities and volumes. However, to fully understand the demands of specific occupational tasks (i.e., clearing a house in military or law enforcement) it is important to include HRV monitoring during the task, in addition to baseline and post-event measures. Since time of day, similar to body position, will significantly influence HRV [38,76], it should be matched across repetitive assessments, where able. Otherwise, comparisons across time-points will be difficult to interpret (i.e., unsure if change is due to training loads or fluctuations in diurnal variation). For example, HF increases at night and decreases during the day, emphasizing the importance of both nocturnal and resting awake readings [26,38]. Figure 3 offers a graphical depiction of the opportunities for HRV measurements throughout the day along with context considerations at each time point.

#### 4.2.1. Establishing Baseline HRV Measures

Establishing a resting state, baseline, HRV is important for assessing overall ANS modulation and cardiac vagal tone. Following the principles of resource theory, baseline HRV identifies the pool of available ANS resources affecting the cardiac cycle [77]. The volume of ANS resources utilized while preparing for, completing, and recovering from psychophysiological demands is related to the imposed workload and functional efficiency to accomplish the task [78]. Comparing the HRV during tasks to HRV before and after tasks can be accomplished through normalization, difference scores, and percentage values for indices of training and recovery loads, respectively. When collecting baseline or resting HRV measures, the supine position is recommended, although sitting or standing may be warranted in field exercises (keeping in mind that methodological consistency is critical) [5,79]. Lastly, confounding factors from external (e.g., temperature, noise, and lighting) and internal (e.g., physical and emotional behaviors leading up to testing) need to be controlled and kept consistent as they will affect HRV and the ability to record “true” baseline values.

**Figure 4:**
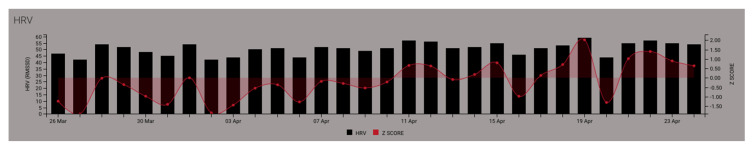
Example of daily heart rate variability (HRV) monitoring of a single individual using a rolling 28-day average to calculate daily Z-scores. Reduced root mean square of the differences in adjacent N-N intervals (rMSSD) and the respective Z-score values below 0 may suggest incomplete recovery, while Z-score values at or above 0 may suggest complete recovery status.

To establish an individual baseline for consistent daily and actionable assessments, HRV measurements should be recorded over a minimum 1–2 week period [46], before making any adjustments to training load or recovery. The utilization of within-individual HRV metric changes are further improved by quantifying rolling 28-day averages [80] and assessing the individual’s deviation from this average. Using the 28-day rolling average, an individual’s change in status is identified as the difference between daily mean (or specific event) HRV and the prior 28-day average HRV. Typically, raw or standard scores (Z-score, percentage change) are compared to rolling average measures. With this approach, acute stress or incomplete recovery states may be denoted by reduced rMSSD as compared to that individual’s 28-day average, as shown in Figure 4 below where Z-score values are less than zero. Conversely, completed recovery could be indicated by maintained or increased HRV [81] shown in Figure 4 where Z-score vales are greater than zero. However, it should be noted that excessive lengths of incomplete recovery can result in an exaggerated increase in parasympathetic modulation, as an attempt to force systemic recuperation efforts [82,83].

#### 4.2.2. Nocturnal and Morning HRV Measures

Nocturnal and morning HRV indicate different psychophysiological processes; however, both inform recovery and readiness status. In long-term monitoring, HRV quantification represents the amount of recovery in relation to average baseline HRV pre-awakening (i.e., during sleep) [84]. Nocturnal HRV, although not completely understood, is commonly used to assess sleep quality, resource recovery, and systemic adaptations [85]. Moreover, assessments pre/post sleep onset and pre/post awakening provide insights to the systemic resources leftover from yesterday’s workload and the subsequent night’s recovery, without and with conscious cognitive processes involved [85]. As stated previously, HRV measurements must be done in a consistent fashion for proper utilization for training load and recovery monitoring. Whether an end user chooses nocturnal or post wake measures, they should be done consistently.

#### 4.2.3. Pre-, During-, and Post-Event HRV Measures

Measuring HRV pre-, intra-, and post-task (e.g., physical or cognitive) may indicate autonomic resources roused in the anticipation of completing a task, the resources required to execute said task, and the rate of resource recovery following completion [35]. HRV responses to events, including training simulations, exercise, or real-world applications, may require a resting HRV measurement 5–10 min prior and a recovery HRV measurement 5–10 min after the event (or at least a known baseline value for difference score calculations). Depending on the circumstances, the first 5 min of a 10 min resting assessment may be discarded as part of an acclimation phase, while the remaining 5 min more accurately represents baseline or recovery HRV. Regardless, the timing of pre- and post- event measures should be similar (i.e., resting measurement 5 min before = resting measurement 5 min after). Furthermore, additional timepoints (e.g., 5–10-, 10–15-, and 15–20 min post-event) may provide further information on the time-course of recovery following the event. The same original temporal duration (e.g., 5-min) should be used for all time blocks and may require parsing into multiple sample windows. Body position (e.g., supine, seated, standing) should also be the same for any pre- and post-measurements.

The modulation of HRV measures can be used to assess various tasks, such as the observe-orient-decide-act cycle of an advanced marksmanship/close quarter battle (CQB) assessment or intense training session, but require more precise time stamping and data parsing compared to pre- and post-task measures [35]. Further, during longer duration sessions, behavioral events (e.g., start/stop, breach, contact, endex, exfil) should be used to determine the context-dependent time windows of specific activities being investigated. Delta scores among time periods and rest/baseline represent the autonomic modulation allocated to process changes in environmental states and accomplish relevant behavioral responses. For example, the difference between baseline and pre-task HRV provides insights to biological priming and arousal/anxiety management, while the difference between baseline to during-task HRV indicates the required ANS resources to achieve the cognitive/motor performances [34,69,86]. As a novice develops expertise, the shift toward automaticity of underlying control processes frees up systemic resources, including HRV [87]. Thus, the aforementioned use of HRV may inform the tasks’ relative difficulty or unfamiliarity, as well as the individual’s level of efficiency and resiliency (i.e., ease of task completion and ability to quickly recover between tasks). For example, smaller changes in HF HRV (i.e., parasympathetic tone), compared to baseline, would suggest greater autonomic resource efficiency and heightened resiliency [11,35,88,89]. This type of HRV perception-action coupling analysis provides a psychophysiological measure of resource allocation, which permits the comparison of performance effectiveness (e.g., resulting outcomes) with resource efficiency (e.g., HRV modulation from baseline) to determine skill automaticity and expertise. Additionally, it enables the tracking of individual progression over time.

#### 4.2.4. Recording 24 h HRV

Continuous measurements over a 24 h period may represent HRV responses to all the various stimuli encountered during typical, daily occupational endeavors. In daily HRV, values below an individual’s normative rolling baseline could indicate incomplete recovery from high training volume, high training intensity, onset of illness, chronic stress, or poor sleep [84]. However, more research is necessary to understand the complexities of daily HRV, which can fluctuate greatly due to diurnal variations in circadian sleep patterns/rhythms, body temperature, metabolism, activity [26]. Still, by encapsulating 24 h HRV recordings a more holistic measure of HRV is permitted. The circadian rhythms may best be accounted for by segmenting the HRV data into daily events, such as nocturnal, morning, training, and occupational events. Nonetheless, a single short duration value should not be used interchangeably with 24 h recordings. The 24 h recordings have been shown to be more reliable than 5 min resting measures, which led some to suggest the use of 24 h recordings for intervention studies [26]. Using the 24 h recording may also allow the quantification of overall ANS responses to a range of stimuli, providing more granularity into wholesome psychophysiological states. However, the execution of collecting 24 h HRV data also requires more buy-in from individuals as they will be tasked with wearing the sensor constantly and ensuring adequate battery charging. Thus, it is necessary to consider whether a method will result in high attrition rates, since large quantities of missing data will not permit reliable, actionable inferences.

## 5. Monitoring Heart Rate Variability (HRV) in Tactical Populations

### 5.1. Relations between HRV and Stress

Stressors experienced by tactical personnel occur in many forms, such as; physical training, deployments, vigilance, emotions, cognitive strain, environmental conditions, lives at stake, low margins for error, and illness [34]. The high-risk nature of operational demands in military environments, particularly special forces [7,90], influence psychophysiological responses of the ANS to environmental conditions [5,31,38,39]. The acute manifestation and chronic accumulation of stress reduces HRV from baseline, which influences performances on physical and mental tasks [34,35,91]. Although maintaining HRV under strenuous loads may indicate preparedness for the task, acute stress responses of reduced HRV in preparation for an event may not necessarily be maladaptive, especially in high performance scenarios that naturally trigger epinephrine responses to improve performance outcomes [91]. Psychological stress, both acute and chronic, usually results in parasympathetic withdrawal, represented by sympathetic dominance or a decrease in HRV (HF and total power) [21]. This response originates from the brain’s perception of the threat or engagement in task workload and is delivered via reduced stimulation of the vagus nerve [92,93]. For example, operators with a high HRV (within and between individuals) are usually better at coping under stressful conditions, expressing greater positive emotions, and performing cognitive tasks more accurately and quickly, whereas operators with low HRV tend to have poor self-regulatory capacity and exhibit by more rigidity and hypervigilance during task responses [44].

### 5.2. Relations between HRV and Physical Occupational Performance and Training Loads

Studies investigating the effects of physical activity on HRV have exponentially increased in recent years [5,16,38,39,94]. In such models, HRV is typically used to detect overtraining or non-functional overreaching (NFOR), which may be detrimental to overall performance and injury risk [5,21,91], as well as stress and recovery [27,46]. Signs of overtraining and NFOR are primarily associated with long-term sympathetic activity (increased LF and suppressed HF) [5,75], however studies also associate chronic overtraining with long-term parasympathetic dominance [5]. Consistent abnormally high or low HRV values may indicate overtraining and suggest a required reduction in training volume (sets x reps); whereas, a reasonably elevated HRV, representing parasympathetic dominance, may indicate a detraining effect and a need for increased training loads [5]. Previous studies reported associations between cardiac autonomic function, as measured by HRV, and aerobic fitness, external training loads, and anabolic hormone concentrations [2,16,24]. Yet, direct comparisons among individuals requires controlling for confounding variables, such as age, gender, and fitness-levels, by comparing individuals to themselves over time or surrogate groups [5,26].

Conventionally, HRV is mainly directed at capturing internal cardiovascular strain resulting from (or required to accomplish) a given external training load. However, for the tactical athlete, quantifying responses to external workloads and cardiovascular homeostatic balance is only one of many actionable insights HRV can afford. Theoretically, running faster and lifting heavier require more resources through PNS withdrawal and SNS stimulation, which influences cardiac output to become more forceful, rapid, and less variable. The scientific literature supports the link between HRV metrics, aerobic adaptations, and general fitness levels [95,96,97], although these relationships may be dictated by the individual’s characteristics and training intensities and volumes. HRV may be useful for understanding the current state of aerobic fitness and monitoring aerobic training adaptations. However, it is important to note that the HRV is mainly a marker of neurocardiac ANS and its direct relation to neuromuscular performances of maximal strength and power output are unclear [97]. Training programs which base workload adjustments on HRV feedback have demonstrated greater increases in countermovement jump and sprinting abilities [98]. Therefore, despite a lack of direct associations between HRV and neuromuscular capabilities, HRV monitoring may improve training program design by prescribing adequate recovery and training volumes. Thus, it is recommended that HRV monitoring is used in conjunction with other monitoring tools including external and internal loads, as well as measures of neuromuscular capacities such as those described in prior literature reviews [99].

### 5.3. Relations between HRV and Cognitive/Motor Skill Performance

Mechanistic underpinnings [11,58,100,101,102,103,104] and applied experimental studies [33,35,69,89,105,106,107,108,109,110] support the use of HRV for assessing, predicting, monitoring, and modulating the cognitive performance domain [59]. Various neural networks obey similar resource theory dynamics and modulate the sympathetic “gas pedal” and parasympathetic “brake.” The prefrontal cortex (PFC) and amygdala, two areas that contribute to neurophysiological control of autonomic tone, are directly responsible for producing key outcomes across several cognitive performance domains [11,12,45,59]. Functional magnetic resonance imaging confirms direct causality between and the vagally mediated HF domain of HRV and PFC activity [58,102,104]. Moreover, the PFC is directly implicated in top-down regulation of several cognitive functions critical to tactical training and operations (e.g., attention/situational awareness, decision making, working memory, and emotion regulation), while the amygdala is at the “heart” of the brain’s fight or flight response and houses much of the arousal and emotional centers (which typically inhibit PFC control) [12,45]. Neural activity covariation suggests cognitive function is directly linked to the medial visceromotor network—the final common pathway through which cognitive function and emotional response influence autonomic control [12,102]. According to this neurovisceral integration model, low HF HRV is indicative of decreased prefrontal inhibitory influences on sub-cortical structures, like the amygdala, and by extension reduced attentional and emotional self-regulation [58,111]. Motor control regions also possess direct and indirect connections with the PFC and amygdala, such that the efficiency of motor skill acquisition, retention, transfer, and resiliency can be indirectly measured through HRV analysis [12,112]. This indicates an imperative role for the autonomic nervous system (and thus HRV) to affect cognitive function across a wide spectrum of behaviors, from classroom/field education to the performance of tactical skills downrange [113].

When presented with a specific task/stimulus, an individual’s HRV response can be altered or vary depending on their knowledge set, skill level, and performance abilities. For the tactical athlete, maintaining emotional (and attentional) control in the face of extreme pressure or danger is vital and influences the entire cognitive performance cascade [12,78]. The ability of an individual to cope with these mental stressors is not only vital to but can alter the psychophysiological mechanisms that control heart rate, and ultimately constrain functionality [12,58,112]. Encountering complex environments that elicit a strong fight or flight response can change self-perception, the current goal-oriented task, and influence attentional/decision making processes, potentially corrupting perception, planning, coordination, and execution of a kinetic response [12,45,114]. Moreover, training and fatigue can also influence within-individual variances across time, such that performance (de)adaptation may be monitored through measuring HRV resource allocation [33,106,115]. For example, during a firefight, a warfighter must pay careful attention to the task at hand, running every bit of relevant visual, auditory, and kinesthetic information through an entire memory registry and decision-making process to rapidly determine the appropriate course of action, then swiftly, yet carefully control their movements to execute the planned response [116,117]. Stress can easily corrupt the entire cascade and this general over-taxation of the system commonly produces a less than desired performance outcome [116,118].

HRV analysis, specifically the fluctuation in HF resource availability and utilization rate/volume, can provide a direct quantification of autonomic efficiency, enabling the measurement of cognitive/motor workload, determination of skill proficiency development across time, and identification of resiliency in the face of other resource demands [33,35,59]. Practically employing HRV in the cognitive performance domain is similar to general physical workload monitoring paradigms, in that, deviations from a resting state or known baseline are compared to time blocks before, during, and after a cognitive or motor demand [69]. Much like physical exercise, decreases in parasympathetic tone (HF HRV) during cognitive/motor skill execution represent resource allocation to accomplish a given goal or task. However, the process is much more temporally sensitive, in that, accurate time-stamping of specific behavioral processes (e.g., anticipation, stimuli presentation, individual responses, post-response recovery) are critical to properly windowing the HRV time-series data [69]. Moreover, while most “physical” HRV paradigms rely on measurement blocks of at least 5 min, many cognitive processes occur on the order of seconds, such that smaller and varying window lengths (e.g., 30, 60, 90 s) may be required to isolate more tactically relevant events (e.g., room-clearing, hand-to-hand combat, calling in a 9-line medevac).

Similar to general resource theory, conservation of autonomic tone (e.g., less disturbance from resting parasympathetic HF) tends to associate with superior performance across numerous facets of perception (attention/emotion regulation, target identification), cognition (working memory, decision making, response inhibition, learning), and action (motor control under duress) [11,35,104,111]. In applied studies, higher resting HRV states (between and within-individual) are most commonly associated with more vagal dominant cognitive control states [11,100], and may indicate a propensity toward superior executive functioning capabilities [59,89,101], while providing resiliency (maintenance of HRV resources from rest to activity/more rapid recovery following) from the negative effects of stress and anxiety [33,69,105,107,119]. Motor performance studies have shown individuals who incur greater deviations from their resting HRV typically demonstrate worse performance on stressful, cognitively challenging motor control tasks [35,106,109]. Additionally, utilizing HRV biofeedback as an educational tool, to promote interoception and self-regulation, has been shown effective at increasing learning rate, reducing performer workload, and increasing performance outcomes [9,10,88,110]. Typically, these paradigms use near-real time HRV markers to help performers identify states during which they may be consuming too many (or the wrong type) of autonomic resources, then measure the affected change from implementing a performance optimization strategy (e.g., mindfulness breathing, cognitive reappraisal).

### 5.4. Recovering to Restore HRV

Much like training load, recovery loads influence HRV responses and may be used to maintain workload capacities over time [120,121]. Proper recovery following training sessions increases the likelihood of long-term desirable adaptations to stress (i.e., training loads), enhancing both workload capacity (i.e., preparedness and resiliency) and well-being [121,122]. Controlling HRV may be as simple as manipulating respiratory processes, such that heart rate increases during inhalation and decreases with exhalation (3,25). During exercise, both heart rate and respiration increase, causing parasympathetic deactivation or withdrawal, thus increasing overall sympathetic tone (25,26). Conversely, deep breathing or tactical breathing techniques can consciously mitigate parasympathetic withdrawal, thus decreasing the effects of sympathetic tone [123].

Post-exercise recovery has gained a great deal of attention in the past decade, leading to a rise in scientific understanding of accelerated recovery techniques to elicit restored autonomic and cardiovascular homeostasis [124,125]. Flotation using R.E.S.T. (Restricted Environmental Stimulation Therapy) has been effective in increasing sleep quality [126] and HRV, specifically the parasympathetic marker HF [127]; thus, being effective in facilitating ANS balance, regardless of pre-float autonomic dominance. Whole-body cryostimulation has an immediate and lasting effect on ANS balance that may be observed via improved HRV [128,129]. For example, 3 min whole-body cryostimulation has increased HF, decreased LF, and decreased LF/HF ratios for upwards of 6 h post-cryotherapy [130,131,132]. Cryotherapy in the evening, after training or competition, has also shown positive effects on sleep quality [129,131,132], which subsequently would improve HRV considering the positive influence of quality sleep hygiene on HRV.

Photobiomodulation (PBM), formerly known as Low-Level Laser Therapy (LLLT) [133], uses red and near-infrared light spectrum wavelengths (600 nm–1100 nm) through light emitting diodes (LED), lasers, or a combination of both, to deeply penetrate the skin and be absorbed by mitochondria of underlying structures (e.g., musculoskeletal tissue) to induce recovery [134,135]. Although limited, the emerging research has shown that whole body PBM may increase sleep quality and the HF of HRV [133,136,137].

Cold Water Immersion consists of 6–20 min immersions in water temperatures below 15 °C (59 °F) [124,125]. The post-exercise effect results in parasympathetic reactivation and sympathetic withdrawal to elicit a restorative effect on autonomic balance and ANS recovery [27,124], resulting in a desirable stress-recovery balance and potentially improving the adaptation to training loads [124].

Many of the aforementioned methods show promise for improving sleep, HRV, and recovery, but require further investigation as to their true efficacy and practicality. However, through the use of a holistic physiological monitoring program, including consistent HRV assessments, it is likely that individual prescription of recovery can be established for the tactical athlete.

## 6. Conclusions

The key parameters of HRV to be concerned with are rMSSD, HF, and TP. Each provides essential information regarding ANS regulation, making HRV a valuable tool to assess the overall health, wellness, and fitness of tactical personnel [5,27]. Training load is affected by intensity and physiological impact of training, which are represented by the changes in HRV during exercise and recovery [79]. HRV is also influenced by cognitive load, physiological and emotional stress, and environment [18,31,123]. In long-term monitoring, it is a non-invasive method to assess the ANS and make appropriate changes to training and recovery [5]. In short-term monitoring, HRV is useful in assessing responses to acute stimuli, such as a physical or cognitive training events [138]. Since, age, body position, and time of day all influence HRV measurements [5,38,52,139,140], HRV should be highly individualized and assessed relative to the individual. Statistically, time periods before, during, and after activity are compared between one another (and as deltas from accurate baselines or rolling averages) to determine resource modulation in relationship to task performance.

Within high performing populations, HRV assessment and monitoring has recently risen in popularity as one method to foster awareness and avoid overtraining or non-functional overreaching [18,53]. The HRV measures may also be used to understand the differences in occupational requirements among occupational specialties. For example, occupational tasks with high demands for concentration and precision, such as paratroopers, dynamic precision shooters, or medics, require significantly more resources and SNS activity than low-stress, office-based occupational tasks [43]. More traditionally, HRV is clinically used to assess both illness and wellness [27,138], primarily cardiac morbidity and the progression of cardiac death, as well as diabetic neuropathy [5,20], all of which should be confirmed by qualified physicians. More recently, HRV metrics are used to garner further understanding of training workload and physical exercise effects on the body [5,20,46,53]. The information gathered is extremely valuable when assessing expertise or preparedness to train, planning training loads, and prescribing the utilization of recovery tools or techniques [5]. For example, those accustomed to greater levels of physical activity and more experienced in given occupational tasks will have improved overall stress management processes, which may indicate improved capacities for handling certain occupational specialties [43]. The likelihood for facilitating greater awareness of one’s own training, recovery, and lifestyle habits, as well as the effect of these habits on their psychophysiology (i.e., HRV), provides another direct benefit of monitoring HRV. Ultimately, HRV measures can be useful for indicating overall health and physical and cognitive preparedness of all tactical personnel, which may be particularly informative for occupational specialties that possess extremely low margins for error in high stress environments (e.g., special forces). Thus, physiological feedback via monitoring HRV would prove useful for addressing the individual’s present capability to handle occupational tasks, be it during selection processes or returning to duty following injury or illness but should not be used to diagnose any pathological conditions.

## Figures and Tables

**Table 1:** Definitions basic time and frequency domain heart rate variability metrics [27,69].

**Time Domains in Short-Term Recordings**			
**Index**	**Definition**	**Interpretation**	**Correlates**
SDNN (ms)	Standard deviation of all R-R intervals	Global quantification of HRV	Total Power
rMSSD (ms)	Root-mean square of successive differences between R-R intervals in a specified time segment	Vagal tone	High Frequency, Parasympathetic activity
**Basic Frequency Domains**			
**Index**	**Definition**	**Interpretation**	**Correlates**
VLF (ms^2^)	Power in the very-low frequency range (<0.04 Hz)	Hormonal factors and peripheral thermoregulation origination	Parasympathetic activity
LF (ms^2^)	Power in the low frequency range (0.04–0.15 Hz)	Baroreflex, arousal	Sympathetic activity, Parasympathetic activity
HF (ms^2^)	Power in the high frequency range (0.15–0.4 Hz)	Cardiopulmonary reflex, cognitive regulatory state, dependent on resource availability and interpretation of environmental demands	Parasympathetic activity
LF/HF	Low frequency/high frequency ratio	Sympathetic-parasympathetic balance (assuming known LF)	Sympathetic activity, Parasympathetic activity
Total Power	Total power in the entire frequency range (<0.4 Hz)	General autonomic resource allocation	Sympathetic activity. Parasympathetic activity
HF/Total Power	High frequency/total power ratio	Proportion of parasympathetic to total autonomic resources	Parasympathetic activity

SDNN, standard deviation of the N-N intervals; RMSDD, root mean square of the differences in adjacent N-N intervals; ULF, ultra-low frequency; VLF, very low frequency; LF, low frequency; HF, high frequency.
